# Serum and 24-hour urinary tests cost-effectiveness in stone formers

**DOI:** 10.1186/s12894-023-01310-w

**Published:** 2023-08-27

**Authors:** Abdolreza Mohammadi, Hiro Farabi, Leila Zareian Baghdadabad, Behzad Narouie, Leonardo Oliveira Reis, Seyed Mohammad Kazem Aghamir

**Affiliations:** 1 Urology Research Center, Tehran University of Medical Sciences, Tehran, Iran; 2 Centre for Evaluation and Methods, Wolfson Institute of Population Health, Barts the London School of Medicine and Dentistry, Queen Mary University of London, Yvonne Carter Building, London, UK; 3 Department of Urology, Zahedan University of Medical Sciences, Zahedan, Iran; 4 Department of Urology, UroScience, Unicamp and Pontifical, State University of Campinas, Catholic University of Campinas, PUC-Campinas, Campinas, Brazil

**Keywords:** Urolithiasis, Metabolic evaluation, Recurrent kidney stone, 24-hour urinary test, Cost-effectiveness

## Abstract

**Objective:**

To assess the routine serum and 24-hour urine tests proficiency in diagnosing the baseline metabolic abnormality of kidney stone formers.

**Methods:**

This study analyzes the routine serum and 24-hour urine tests proficiency in diagnosing the baseline metabolic abnormality of kidney stone formers. The sensitivity and specificity, false positive, and negative results of the tests are extracted from diagnostic kits used in the laboratories of the target community. To accurately infer the results, a simulation based on 1000 people was used through 22 standard laboratory tests (Additional File 2), including calcium, oxalate, phosphate, uric acid, sulfate, potassium, sodium, citrate, and magnesium in 24-hour urine; and calcium, creatinine, Vit D, uric acid, and intact parathyroid hormone (PTH) in serum. The incremental cost-effectiveness ratio (ICER) was calculated and compared for each diagnostic test versus other diagnostic tests according to the incremental cost required for correct diagnoses of stone causes.

**Results:**

Urinary uric acid, citrate, and serum potassium constitute the cost-effectiveness boundary curve in this study. This means that other diagnostic tests are not cost-effective compared to these three tests in terms of indexing at least one item of cost and effectiveness. The ICER index for each correct diagnosis with the urinary uric acid test was $ 1.25 per diagnosis, the most cost-effective test compared to serum potassium and urinary citrate.

**Conclusion:**

The simplified blood and 24-hour urine metabolic evaluation, including urinary uric acid, citrate, and serum potassium, constitute the cost-effectiveness boundary curve. The most cost-effective test was urinary uric acid measurement.

**Supplementary Information:**

The online version contains supplementary material available at 10.1186/s12894-023-01310-w.

## Introduction

Urolithiasis is among the most common urologic diagnoses globally, with considerable burden and cost on healthcare systems worldwide. The most relevant risk factors include diet and lifestyle trends, common diseases such as diabetes and obesity, and global warming [1]. The worldwide prevalence, incidence, and composition of calculi vary according to geographical area, with prevalence ranging from 7 to 13% in North America, 5–9% in Europe, and 1–5% in Asia [2]. The recurrence rate without preventive treatment is approximately 10% at one year, 33% at five years, and 50% at ten years. Kidney stone recurrence rates vary by the underlying metabolic cause. Eligible patients including recurrent active stone formers and single-stone formers with individual risk factors, are considered for full metabolic evaluation that relies on 24-hour urine collection to diagnose metabolic abnormalities and future pharmacologic therapy to prevent a recurrence [3]. Nephrolithiasis is currently the most expensive urological condition, estimated to cost the healthcare system more than $10 billion per year. As well as anticipated population growth, current projections estimate costs due to stone disease to rise by $1.24 billion per year by 2030 [4]. The social impact is represented by its sequelae of renal colic, loss of work, the need for medical care, hospitalization, and urological intervention. The renal function may be affected, and mild to moderate chronic renal insufficiency is expected to develop in up to this 20% of these patients [5]. The initial evaluation for most first-time stone patients includes urinalysis, urine culture, and blood profile including calcium, phosphorus, uric acid, and serum creatinine analysis. The charges for this evaluation range from $227 to $269, depending on whether urine cultures are indicated [6]. More than 10% of patients initially evaluated in the emergency department (ED) require a return visit in 30 days, further exacerbating costs and reflecting high patient morbidity [7]. So, 24-hour urine collection is essential for the prevention of recurrent stones in high-risk patients, but there are some difficulties in collecting urine samples including the cost, and being time-consuming. The delayed collection will increase resource use and prolong hospital bed occupancy. Poor quality samples lead to missed diagnoses, unnecessary follow-up, and investigations. Current guideline recommendations for urine collection methods do not incorporate cost-effectiveness evidence. Given the very high recurrence rates, treatment aimed at preventing stone formation is critical to diminishing the morbidity and costs associated with the disease [8]. Therefore, we designed a decision tree model to evaluate the cost-effectiveness of serum and urinary tests in the management strategies of stone formers and identify the most efficient tests.

## Methods

### Study design

Data were provided by Persian Registry for Stones of Urinary System (PERSUS). This study is an economic evaluation that analyzes the normal serum and 24-hour urine tests proficiency in diagnosing the baseline metabolic abnormality of kidney stones from the provider’s perspective. The values for serum and 24-hour urine parameters are consistent with the guidelines established by the American Urological Association (AUA) and the European Association of Urology (EAU).

All patients signed the written informed consent, and the study was approved by the Tehran University of Medical Sciences ethical committee *(IR.TUMS.MEDICINE.REC.1400.663).* The target population that was included in the study were patients with recurrent stones and high-risk first-time stone-formers that referred to the hospital for serum and metabolic 24-hour urine tests. The main aim of these tests was a diagnosis of the etiologic abnormalities of urolithiasis. The study was simulated for a hypothetical group of 1000 people. Decision analysis tree (Additional File 1) and Treeage 2011 software were used to analyze the cost-effectiveness of kidney stone diagnosis tests in patients, focusing on every test recommended in the main guidelines.

### Targeted outcomes

The primary outcome of this study is the effectiveness of discrete diagnostic tests used to investigate the underlying metabolic abnormalities that could result in stone formation. The sensitivity and specificity, false and true positive, and negative results of the tests are extracted from diagnostic kits used in the laboratories of the target community. A simulation based on 1000 people was used to infer the results more accurately. Based on this, 1000 people with a complaint of kidney stones are examined through 22 standard laboratory tests (Additional File 2). The recommended and most commonly evaluated metabolites are calcium, oxalate, phosphate, uric acid, sulfate, potassium, sodium, citrate, and magnesium in a 24-hour urine test; and calcium, creatinine, serum uric acid, Vit D3, and intact parathyroid hormone (PTH) in serum. The results will demonstrate the possible underlying causes of the stone formation.

Suppose the person has a kidney stone and the “metabolic evaluation” tests for underlying etiology were positive. In that case, the result is truly positive, and the cause of the person’s stone is the item examined in the metabolic test. Likewise, if a person has a kidney stone, but the “cause of the stone” test is negative, the results will be false negatives (sensitivity-1) [9]. True negatives and positive tests are considered efficient in diagnosing the cause of kidney stones. In contrast, false positives and negative tests are considered incorrect diagnoses. Both groups of results (true positive and negative, false positive and negative) were used as the result of each diagnostic technique in the final knob of the decision tree.

### Cost

The costs included direct medical expenses that were extracted from the receipt provided to the patients. All costs spent to diagnose the underlying metabolic cause of the stone formation were measured in rials (Iran’s common currency) and converted to dollars (1 dollar = 280,000 rials, conversion rate in 2022). The costs contained in the patient receipt included all costs of sampling, examination, analysis of samples, and other laboratory overhead costs.

### Cost-effectiveness analysis

A cost-effectiveness analysis was performed based on each diagnostic metabolic test (serum or 24-hour urine). The incremental cost-effectiveness ratio (ICER) was calculated and compared for each diagnostic test versus other tests according to the incremental cost required for correct diagnoses of stone causes.

The CER ratio was calculated based on the following

$$ICER=\frac{{Cost}_{Test1}-{Cost}_{Tes2}}{{Effectveness}_{Test1 }-{Effctiveness}_{Test2}}$$

ICER is a tool that can assess the economic evaluation of an intervention (for example, a particular drug) compared to other interventions. ICER shows how much it costs to obtain an additional unit of health benefits from one intervention to another. The cost-effectiveness of an intervention depends on its relationship to the maximum willingness to pay for an outcome or, as the saying goes, the ICER threshold. If the cost-effectiveness of the intervention is less than the threshold, the intervention is considered cost-effective. The intervention is not cost-effective if it is above the ICER threshold [10]. The risk of recurrence is roughly 50–80%, depending on the type of stone and time from the first episode of urolithiasis, unless secondary prevention is started. Risk-adapted secondary prevention reduces this risk to 10–15% [11].

### Sensitivity analysis

Sensitivity analysis refers to changing one or more important parameters and their effect on the model’s outcome. For sensitivity analysis, indeterminate parameters that are exogenous and beyond the researcher’s control were identified using tornado diagrams. Finally, using one-way sensitivity analysis, the effect of the parameters on the results was determined [10]. Since different laboratories might use other kits for diagnosis, the precise evaluation of tests’ cost-effectiveness and stability is essential. One-way sensitivity analysis was used. In a one-way sensitivity analysis, each parameter value is evaluated independently. The analysis is rerun by using a range of assumed values for the one-parameter while keeping all of the others fixed at their base-case values [12]. Sensitivity analysis was performed using a 10% range of the sensitivity and specificity rate of tests. Furthermore, the prevalence of kidney stones was evaluated as a parameter with uncertainty with a 10% change in the sensitivity analysis.

### Data sources

Epidemiological information and probabilities of each branch of the decision-analysis tree were collected from diagnostic kits used in the laboratory, scientific sources, and convincing national and international articles. This information includes the prevalence of kidney stones and diagnostic methods’ sensitivity and specificity (Table 1).

**Table 1:** Diagnostic tests cost, sensitivity, and specificity

Diagnostic Test	Cost (USD)	Sensitivity	Specificity	Reference (Senstivity&Specifity)
**Serum Chloride**	1.01	0.82	0.89	Linda Shavit1,2, Lucia Chen1, Fayha Ahmed3, Pietro Manuel Ferraro4, Shabbir Moochhala1, Steven B. Walsh1, Robert Unwin. Selective screening for distal renal tubular acidosis in recurrent kidney stone formers: initial experience and comparison of the simultaneous furosemide and fludrocortisone with the short ammonium chloride test. *Nephrology Dialysis Transplantation*, Volume 31, Issue 11, November 2016, Pages 1870–1876
**Urinary Calcium**	1.18	0.57	0.68	Rossi MA, Singer EA, Golijanin DJ, Monk RD, Erturk E, Bushinsky DA. Sensitivity and specificity of 24-hour urine chemistry levels for detecting elevated calcium oxalate and calcium phosphate supersaturation. Canadian Urological Association Journal. 2008 Apr;2(2):117
**Urinary Sodium**	5.04	1.00	0.82	WolfgangWeger, Peter Kotanko, MartinWeger, Hannes Deutschmann and Falko Skrabal. Prevalence and characterization of renal tubular acidosis in patients with Osteopenia and osteoporosis and in non-porotic controls. Nephrol Dial Transplant (2000) 15: 975–980
**24 H Urine ph**	0.98	1.00	0.62	Adrian Rossi, MD;* Eric A. Singer, MD;* Dragan J. Golijanin, MD;* Rebeca D. Monk, MD;† Erdal Erturk, MD;* David A. Bushinsky, MD†. Sensitivity and specificity of 24-hour urine chemistry levels for detecting elevated calcium oxalate and calcium phosphate supersaturation CUAJ 2008;2(2):117 − 22.
**Urinary Oxalate**	2.63	0.59	0.65	Rossi MA, Singer EA, Golijanin DJ, Monk RD, Erturk E, Bushinsky DA. Sensitivity and specificity of 24-hour urine chemistry levels for detecting elevated calcium oxalate and calcium phosphate supersaturation. Canadian Urological Association Journal. 2008 Apr;2(2):117
**Serum IPTH-CLIA**	5.72	0.90	0.77	Hyperparathyroidism (primary): diagnosis, assessment, and initial management Evidence review for Diagnostic Tests NICE guideline NG132 Diagnostic evidence review May 2019
**Serum Sodium Na**	1.08	0.82	0.31	Bruno Madeo,1 Elda Kara,1 Katia Cioni,1 Silvia Vezzani,1 Tommaso Trenti,2 Daniele Santi,1,3 Manuela Simoni,1,3,4 and Vincenzo Rochira. Serum Calcium to Phosphorous (Ca/P) Ratio Is a Simple, Inexpensive, and Accurate Tool in the Diagnosis of Primary Hyperparathyroidism. JBMR1 Plus, Vol. 2, No. 2, March 2018, pp 109–117
**Serum Uric Acid**	0.93	0.98	0.87	WolfgangWeger, Peter Kotanko, MartinWeger, Hannes Deutschmann and Falko Skrabal. Prevalence and characterization of renal tubular acidosis in patients with Osteopenia, osteoporosis, and non-porotic controls. Nephrol Dial Transplant (2000) 15: 975–980
**Urinary Uric Acid**	0.93	0.79	0.92	Adrian Rossi, MD;* Eric A. Singer, MD;* Dragan J. Golijanin, MD;* Rebeca D. Monk, MD;† Erdal Erturk, MD;* David A. Bushinsky, MD†. Sensitivity and specificity of 24-hour urine chemistry levels for detecting elevated calcium oxalate and calcium phosphate supersaturation CUAJ 2008;2(2):117 − 22.
**Urinary Magnesium**	1.38	0.59	0.77	Adrian Rossi, MD;* Eric A. Singer, MD;* Dragan J. Golijanin, MD;* Rebeca D. Monk, MD;† Erdal Erturk, MD;* David A. Bushinsky, MD†. Sensitivity and specificity of 24-hour urine chemistry levels for detecting elevated calcium oxalate and calcium phosphate supersaturation CUAJ 2008;2(2):117 − 22.
**Urinary Potassium**	1.08	0.8060	0.8570	WolfgangWeger, Peter Kotanko, MartinWeger, Hannes Deutschmann and Falko Skrabal. Prevalence and characterization of renal tubular acidosis in patients with Osteopenia, osteoporosis, and non-porotic controls. Nephrol Dial Transplant (2000) 15: 975–980
**Urinary Citrate**	7.86	0.8600	1.0000	WolfgangWeger, Peter Kotanko, MartinWeger, Hannes Deutschmann and Falko Skrabal. Prevalence and characterization of renal tubular acidosis in patients with Osteopenia, osteoporosis, and non-porotic controls. Nephrol Dial Transplant (2000) 15: 975–980
**Serum Calcium**	1.18	0.9	0.99	Bruno Madeo,1 Elda Kara,1 Katia Cioni,1 Silvia Vezzani,1 Tommaso Trenti,2 Daniele Santi,1,3 Manuela Simoni,1,3,4 and Vincenzo Rochira. Serum Calcium to Phosphorous (Ca/P) Ratio Is a Simple, Inexpensive, and Accurate Tool in the Diagnosis of Primary Hyperparathyroidism. JBMR1 Plus, Vol. 2, No. 2, March 2018, pp 109–117
**Serum Potassium K**	1.08	0.96	0.99	Xilian Qiu1,*, Chunyong Liu2,*, Yuqiu Ye3,*, Huiqun Li3, Yanbing Chen4, Yongmei Fu3, Zhenjie Liu2, Xianzhang Huang2, Yunqiang Zhang5, Xueyuan Liao5, Hongyong Liu5,*, Wenbo Zhao3 and Xun Liu. The diagnostic value of serum creatinine and cystatin c in evaluating glomerular filtration rate in patients with chronic kidney disease: a systematic literature review and meta-analysis. Oncotarget, 2017, Vol. 8, (No. 42),
**Serum Vit D**	4.16	0.91	0.95	Hyperparathyroidism (primary): diagnosis, assessment, and initial management Evidence review for Diagnostic Tests NICE guideline NG132 Diagnostic evidence review May 2019
**Urinary Cystine**	1.33	0.95	0.72	Andreassen KH, Pedersen KV, Osther SS, Jung HU, Lildal SK, Osther PJ. How should patients with cystine stone disease be evaluated and treated in the twenty-first century? Urolithiasis. 2016 Feb;44:65–76.
**Urinary Phosphate**	3.68	1.00	0.94	Rossi MA, Singer EA, Golijanin DJ, Monk RD, Erturk E, Bushinsky DA. Sensitivity and specificity of 24-hour urine chemistry levels for detecting elevated calcium oxalate and calcium phosphate supersaturation. Canadian Urological Association Journal. 2008 Apr;2(2):117

## Results

Our model is intended for a group of 1000 people (the simulation results are in Additional File 2). The cost of each test, the theoretical cost of each correct diagnosis, and its effectiveness are summarized in Table 2.

**Table 2:** Test effectiveness and the average cost of correct diagnosis

Diagnostic test	اeffectiveness	Cost for 1000 tests (USD)	Effectiveness for 1000 tests	The average cost for each real positive test (USD)
**Serum Chloride**	17%	1012.05	172	5.90
**Urinary Calcium**	67%	1177.12	669	1.76
**Urinary Sodium**	83%	5041.19	834	6.04
**24 H Urine pH**	66%	981.83	655	1.50
**Urinary Oxalate**	64%	2627.76	642	4.10
**IPTH-CLIA**	78%	5720.52	780	7.33
**Sodium Na**	35%	1079.48	352	3.06
**Serum Uric Acid**	88%	926.81	877	1.06
**Urinary Uric Acid**	91%	926.81	910	1.02
**Urinary Magnesium**	76%	1377.81	759	1.81
**Urinary Potassium**	85%	1079.48	853	1.26
**Urinary Citrate**	99%	7862.17	989	7.95
**Serum Calcium**	99%	1176.88	987	1.19
**Serum Potassium K**	99%	1079.48	987	1.09
**Serum Vit D**	95%	4159.29	948	4.39
**urinary Cystine**	74%	1334.59	738	1.81
**Urinary Phosphate**	95%	3678.57	948	3.88

Table 3 shows the most cost-effective tests for informed decision-making, which might be used in groups according to the payer’s financial resources.

**Table 3:** Tests cost-effectiveness for informed decision-making

Diagnostic tests	Cost per diagnosis (USD)
**Urinary Uric Acid**	1.24
**Urinary Potassium**	1.63
**Serum Potassium K**	6
**Serum Calcium**	1.39
**Urinary Cystine**	2.60
**Urinary Magnesium**	2.41
**Urinary Phosphate**	46
**Urinary Citrate**	129.5

**Fig. 1:**
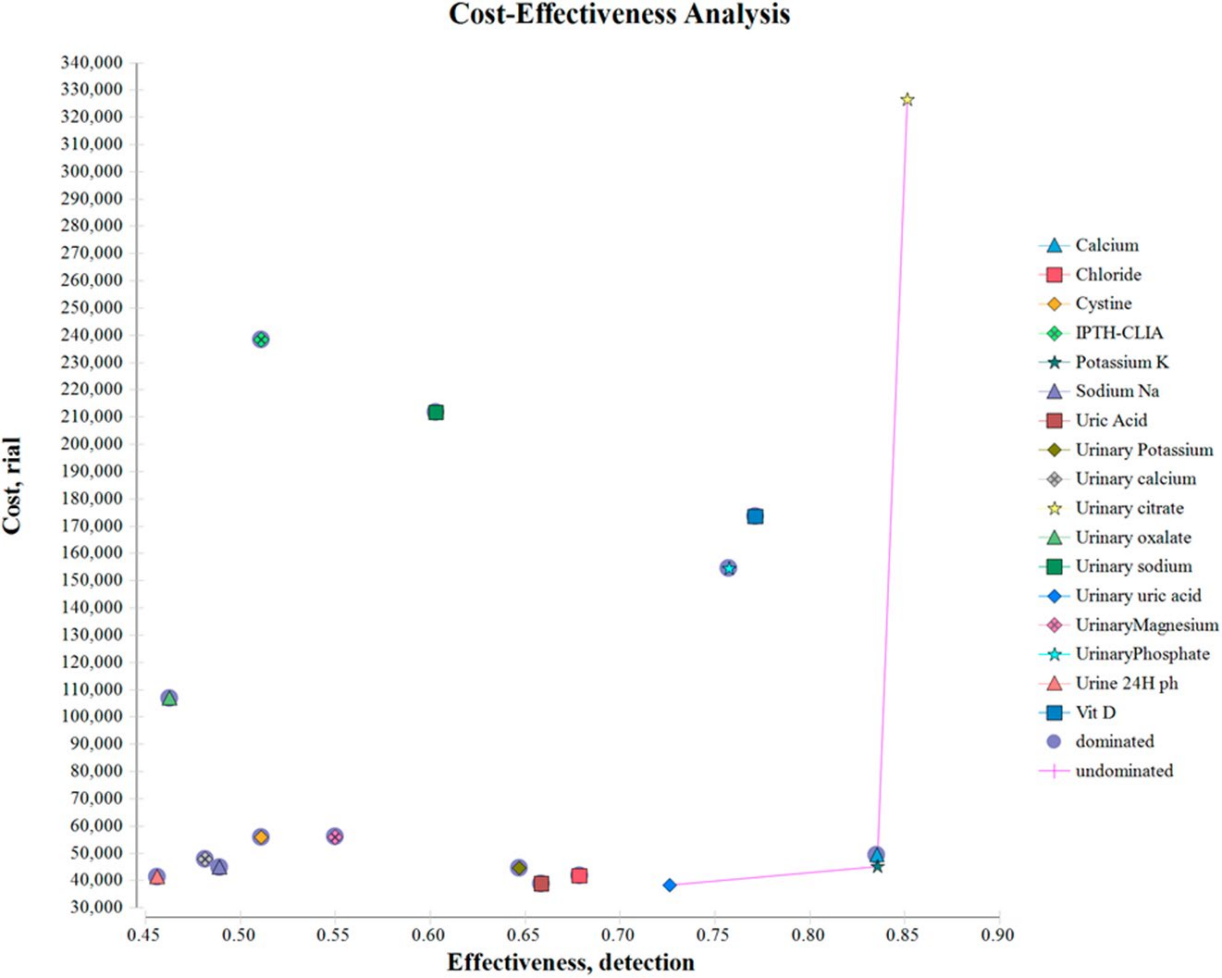
Cost-effectiveness diagrams of kidney stone diagnostic tests

Figure 1 shows that three tests to diagnose the underlying cause of kidney stones, including urinary uric acid, serum potassium (Potassium K), and urinary citrate, constitute the cost-effectiveness boundary curve in this study (Group 1). This means that other diagnostic tests are less cost-effective than these three tests in terms of indexing at least one item of cost and effectiveness.

The mentioned items changed as follows: The ICER index for each correct diagnosis with the urinary uric acid test was $ 1.25 per diagnosis, the most cost-effective test compared to serum potassium (K) and urinary citrate, which were on the cost-effectiveness boundary. Therefore, the urinary uric acid test is the most effective test to diagnose kidney stones cause. The ICER index for potassium K and Urinary citrate is $ 6 and $ 129.5 for one diagnosis, respectively.

The second top-ranked group of cost-effective tests is urinary uric acid, serum potassium (K), serum calcium, and urinary citrate (Group 2), especially in conditions we portend with resource restrictions. More tests might be offered if the payer’s financial resources are more flexible. Therefore, compared to incremental costs and incremental effectiveness and the four tests mentioned in the previous step, the urinary magnesium and phosphate tests are cost-effective and constitute the third superior group (Group 3). Group 4 includes urinary uric acid, serum potassium, serum potassium K, serum calcium, serum cystine, urinary magnesium, urinary phosphate, and urinary citrate in terms of unlimited resources.

## Discussion

Urolithiasis is an increasing global problem, mainly due to industrialization, climate, and lifestyle changes, with a significant recurrence rate. A comprehensive workup, including medical history, physical examination, basic urine, blood analysis, and radiological studies, is recommended in all patients with urolithiasis. Essential metabolic evaluations comprise the serum creatinine, calcium, sodium, potassium, uric acid, and PTH in patients with an increased serum calcium level [13, 14].

If stone fragments are collected during surgery, they should be sent for analysis [15]. In recent years the main focus is targeted medical therapy according to the underlying metabolic abnormalities that predispose to stone formation. The main aim of individualized evaluations is to exclude underlying metabolic abnormalities and start stone-specific recurrence prevention [16]. Several studies regarding the cost-effectiveness of different treatment modalities in stone management are available [17–20]. However, to our knowledge, this study is the first economic evaluation of the cost-effectiveness of serum and 24-hour urine tests recommended in the main guidelines. A study by Lotan et al. evaluated the cost-effectiveness of nutritional measures and medical therapy (empiric or directed) according to the complete metabolic assessment of recurrent stone formers. They concluded that conservative dietary measures are more cost-effective than medical drug therapy [21]. Another study by these authors evaluated the cost-effectiveness of primary prevention in urolithiasis. They concluded that primary prevention could be cost-effective for a population with high urolithiasis frequency (low cost and moderately effective), however, some diet modifications and subsequent urinary pH in patients with uric acid kidney stones does not change with dietary intake [22].

Strohmaier et al. evaluated the cost-effectiveness of dietary measures compared to medical therapy. They conclude that if stone incidence is more than one stone per patient per year, medical treatment will be more cost-effective than dietary measures [23].

The European Urology Association (EAU) and American Urological Association (AUA) guidelines on urolithiasis mentioned the following tests for full metabolic evaluation: serum evaluation including creatine, uric acid, calcium, sodium, potassium, C-reactive protein, chloride, intact PTH, and 24-hour urine evaluation including calcium, oxalate, citrate, uric acid, phosphate, sulfate, sodium, potassium, cystine, magnesium, and PH [24].

The main reason for these tests is the recognition of specific metabolic abnormalities in 24-hour urine that help urologists reduce recurrent stones with individualized diet and medical therapy. However, the results may be normal in stone formers, and abnormal in non-stone formers. In a study by Eisner et al. on differences between 24-hour urine abnormalities in first-time stone formers and recurrent stone formers, they concluded that the probability of having a single abnormality of 24-hour urine composition was similar between the two groups (83.1% for first-time vs. 88.8% for recurrent) [25].

A study by Chan et al. on eighty pediatric patients with urolithiasis found that a restricted metabolic evaluation, including the calcium, oxalate, citrate, and urinary volume in a 24-hour urine test, is sufficient to recognize the most frequent metabolic abnormalities [26]. Also, Oguz et al. identified hypercalciuria, hypomagnesuria, and hypocitraturia as the most critical risk factors for urolithiasis in 257 adults and pediatric patients with urinary stones [27].

Eyre et al. evaluated the utility of serum calcium, parathyroid hormone (PTH), urate, chloride, bicarbonate, potassium, and phosphate in screening metabolic abnormalities in 709 renal stones formers and revealed elevated serum calcium levels in 2.3% of patients. They concluded that serum calcium measurement alone is sufficient in most patients with urolithiasis [28].

An international cost survey by Chandhoke et al. revealed that in acute renal colic management, the metabolic assessment and directed medical therapy were only cost-effective when at least one stone episode every three years [29]. Ghanem et al. evaluated 457 patients with urolithiasis, and a low urine volume was the only finding in 24-hour urine metabolic workup in first stone former compared to recurrent stone formers. They recommended that metabolic abnormalities be evaluated only in recurrent stone formers [30].

Our study has distinguished properties. First, it could be supposed that by selecting and replacing from suggested Group 1 with Group 4, more tests will be cost-effective due to access to resources and will be economically viable. In such a way that in the most limited state of allocation resources, little metabolic evaluation using little tests including urinary uric acid, serum potassium (K), and urinary citrate might be done, and with fewer restrictions on funding and resources, packages with more tests can be offered. No studies have been found to evaluate the effectiveness of limited metabolic evaluation, but there is evidence of limited testing effectiveness for assessing the cause of stones. In the best situation of allocating resources, eight tests are more cost-effective among the 17 mentioned in the main guidelines, including urinary uric acid, potassium, magnesium, phosphate, citrate, serum potassium, calcium, and cystine. In the resource restriction, serum potassium is dominant to urinary potassium, and cystine is dominant to serum calcium, so six tests will be cost-effective. In even more limited circumstances, the two urinary phosphate and citrate tests should be excluded.

Sensitivity analysis with a 10% change in the sensitivity and specificity of the kits used to diagnose the cause of the stone showed that the results did not change. Therefore, the cost-effectiveness is not vulnerable to the sensitivity and specificity variance among available tests.

After sensitivity analysis, interestingly, in the special tests’ categorization to present in conditions with different financial resources, only three group tests will remain, and more difficulties will be removed in the initial step of comparing incremental costs and incremental effectiveness. In addition, in the third group, the position of the tests will change in terms of cost-effectiveness. In a way, urinary magnesium is not more economical in this group than before, and urinary potassium, which was not previously economical, will be included. Therefore, it could be concluded that the obtained results are sensitive to the prevalence of kidney stones, and the results will change in different regions with a different majority.

Depending on stone incidence, type of insurance, and cost of interventional modalities, medical and surgical therapy cost-effectiveness is changeable in different countries, with a trend to more effective stone prevention medical therapy in low-income countries. Since the targeted medical treatment based on the 24-hour urine findings could result in a 50% decrease in stone recurrence, we need to focus more on simplifying the metabolic evaluation and improving patient compliance.

It was necessary to determine the cost-effectiveness index to provide an informed decision on the willingness to pay for the correct diagnosis. Evaluating the ICER index according to the cost-effectiveness threshold, the primary outcomes of the current study were considered the number of accurate diagnoses in each test. In our unpublished study on PSA screening tests, the willingness to pay was 96 dollars. Therefore, the cost-effectiveness of all three tests at the border of the cost-effectiveness curve (Group 1) can be confirmed. Once metabolic tests are not substitutes for each other, the main purpose of these steps is to report the most cost-effective tests, especially in terms of resource constraints and where resource allocation efficiency is essential, so we expand the analysis one step further than the most cost-effective test for diagnosing the underlying cause of kidney stones.

Finally, the serum blood test and 24-hour urine metabolic test have several restrictions, including inadequate sample gathering, the need for repeat tests, the difficulty of analysis, and different laboratory references. Due to the multifactorial nature of the stone formation, it is tough to contribute the findings in the metabolic evaluation as the only factor of the stone formation. Our results shed light on informed decision-making, simplifying the metabolic evaluation in recurrent stone formers. Stakeholders and policymakers need to take these results into account when deciding on healthcare budget allocation, as the management of stones can be expensive. One of our study’s limitations is that our model makes assumptions based on previously issued reports. We recommend new models that take into account the efficacy of various components in 24-hour urine examinations.

## Conclusion

Using cost-effectiveness analysis, four different test groups can be distinguished in the limited metabolic evaluation of kidney stone patients. The simplified blood and 24-hour urine metabolic evaluation constitutes the cost-effectiveness boundary curve, including urinary uric acid, serum potassium, and urinary citrate. The most cost-effective test, unchanged in the cost-effective analysis model, was urinary uric acid measurement. Stakeholders and policymakers need to take these results into account when deciding on healthcare budget allocation, as the management of stones can be expensive.

### Electronic supplementary material

Below is the link to the electronic supplementary material.

Additional File 1: Decision analysis treeto analyze the cost-effectiveness tests

Additional File 2: Simulation of 22 standard laboratory test

## Data Availability

The datasets generated and analyzed during the current study are available from the corresponding author upon reasonable request.
