# Supplementary material for: Serum and 24-hour urinary tests cost-effectiveness in stone formers
Source: BMC Urol. 2023 Aug 27;23:141. doi: 10.1186/s12894-023-01310-w (PMC10464256; doi:10.1186/s12894-023-01310-w)
Supplement: Supplementary file 1 — Additional File 1: Decision analysis treeto analyze the cost-effectiveness tests [file 12894_2023_1310_MOESM1_ESM.docx]

| Serum biochemical | **Sensitivity %** | Specificity% |
| --- | --- | --- |
| Chloride | 82.2 | 88.50 |
| Creathine | 80 | 87 |
| IPTH-CLIA | 85 | 88 |
| Sodium Na | 82.5 | 31.1 |
| Uric Acid | 97.8 | 86.80 |
| Urea | 75 | 64.8 |
| Calcium | 60 | 67.3 |
| Potassium K | 85 | 77 |
| Vit D | 87.8 | 87.2 |
| Cystine | 72 | 95 |
| urinary variables(24-h excretion (mmol/day) | **Sensitivity %** | Specificity% |
| Urinary calcium | 57.1 | 67.7 |
| Urinary sodium | 56.3 | 56 |
| Urine 24H ph | 100 | 62.5 |
| Urinary oxalate | 43.70 | 64.6 |
| Urinary creatinine | 84 | 76 |
| Urinary uric acid | 52.9 | 51.00 |
| UrinaryMagnesium | 59. | 77.4 |
| Urinary Potassium | 80.6 | 85.7 |
| Urinary citrate | 45.4 | 50 |
| UrinaryPhosphate | 80 | 97 |
| Urinary urea | 89.2 | 96.7 |
| Total volume | 90.9 | 97.2 |
