# Supplementary figures and images for: Serum and 24-hour urinary tests cost-effectiveness in stone formers
Source: BMC Urol. 2023 Aug 27;23:141. doi: 10.1186/s12894-023-01310-w (PMC10464256; doi:10.1186/s12894-023-01310-w)

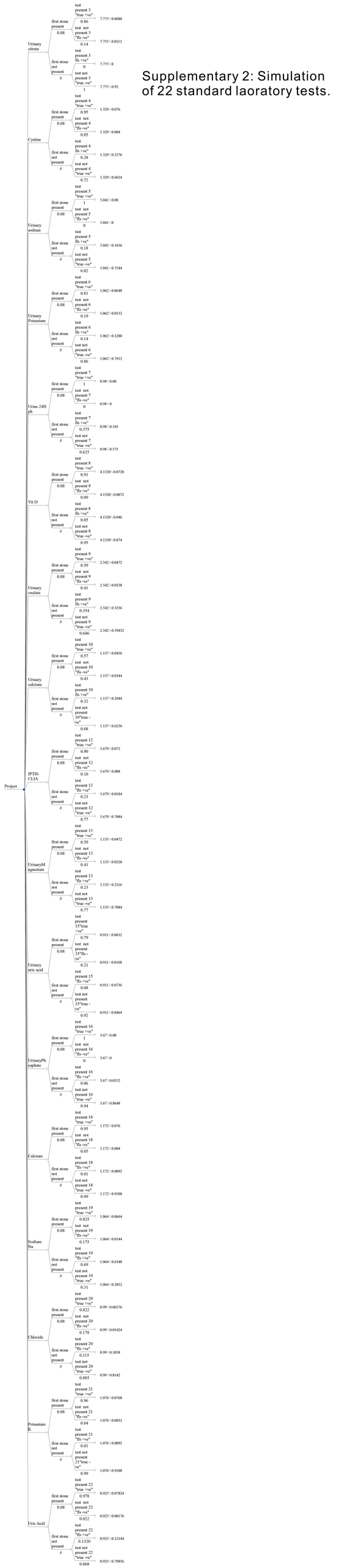

Supplement: Supplementary file 2 — Additional File 2: Simulation of 22 standard laboratory test [file 12894_2023_1310_MOESM2_ESM.jpg]
